# Role of Notch1 Signaling Pathway in Small Cell Lung Carcinoma

**DOI:** 10.30699/IJP.2024.2013339.3184

**Published:** 2024-10-30

**Authors:** Mohammed Ahmed Charbat, Yousuf Hafez Abdulhalim, Mohammed Abdullatif Alrabeei, Wael Abdo Hassan

**Affiliations:** 1 *College of Medicine, Sulaiman Al-Rajhi University, Al-bukayriyah, Kingdom of Saudi Arabia*; 2 *Faculty of Medicine, Department of Pathology, Suez Canal University, Ismailia, Egypt*; 3 *Department of Basic Sciences, Sulaiman Al-Rajhi University, Al-bukayriyah, Kingdom of Saudi Arabia *

**Keywords:** Molecular Pathway, Notch1 signaling; SCLC, Small cell lung carcinoma; Targeted therapy, Tumor suppressor

## Abstract

Lung cancer is the leading cause of cancer-related death around the globe. It is generally divided into small-cell and non-small-cell lung carcinomas. Small-cell lung cancer (SCLC) is a malignant tumor characterized by rapid growth, high metastatic potential, and a frequent rate of relapse after chemotherapy. All the features may worse aggressiveness of this cancer and increase the possibility of unsuccessful therapeutic attempts. Notch1 signaling is a crucial molecular pathway in the regulation of many cellular functions, including cell-cell communication and gene regulation. Moreover, it was proposed previously that Notch1 might be oncogenic in various types of cancer, but the question arises as to why many SCLC cell lines do not express this pathway. This review aims to explore the role of this complex pathway in SCLC in both vitro and vivo studies and whether it has a tumor-promoting or suppressive effect. After an extensive literature review, it was found that the expression of Notch1 signaling in SCLC reduces its proliferative ability while promoting increased cell apoptosis. Furthermore, it reduces cell motility, invasion, and metastatic ability and enhances cell-cell adhesion by inhibiting epithelial-mesenchymal transition (EMT). Furthermore, it contributes to cell chemo-resistance by altering multidrug resistance-associated protein-1 (MRP-1), demonstrating an overall tumor-suppressive effect. Given these findings, induction of Notch1 using histone deacetylase inhibitor (HDACi) may be a potential future therapeutic strategy for SCLC management. Nevertheless, the effect of such a sophisticated signaling pathway in tumor carcinogenesis can’t be generalized to all human cancers, and further studies are needed to better tailor therapeutic plans based on the specific cellular context.

## Introduction

Worldwide, in 2018, there were 2.09 million cases of lung cancer, resulting in 1.76 million deaths. This makes it the leading cause of cancer-related deaths around the globe (1). Lung cancer is histologically divided into two main categories: (1) small cell lung carcinoma (SCLC), (2) non-small cell lung carcinoma (NSCLC), which is defined as any type other than SCLC, e.g., adenocarcinoma (ADC), large-cell lung carcinoma (LCLC), and squamous cell carcinoma (SCC) (2). The long-term use of tobacco accounts for up to 85% of lung cancer cases (3). Other common causes include genetic components, exposure to asbestos, radon gas, or other air pollution (4,5). patients usually present with coughing, shortness of breath, weight loss, and chest pain, among other symptoms (6). The long-term outcomes of lung cancer treatment are not straightforward, and factors such as type of cancer, the patient’s overall health, and the current stage of cancer before treatment can affect the final outcome (7). In most cases, surgical resection is the primary treatment for NSCLC, while SCLC usually responds better to chemotherapy and radiation (7).

SCLC is a type of lung cancer composed of relatively small, poorly differentiated tumor cells with scant cytoplasm that often arises from neuroendocrine (NE) cells. It accounts for nearly 20% of the cases and is characterized by rapid growth and metastasis (2, 8). These features make SCLC one of the most malignant types of cancer, with a high rate of relapse after chemotherapy (9). Moreover, due to the SCLC neuroendocrine (NE) nature, patients with this tumor may develop serious para-neoplastic syndromes, like Cushing syndrome (increased ACTH), the syndrome of inappropriate antidiuretic hormone (SIADH), and neuromuscular syndromes, such as Lambert-Eaton myasthenic syndrome (LEMS) (production of autoantibodies against voltage-gated calcium channels in the presynaptic neuronal cell membrane). This makes SCLC one of the most aggressive types of lung cancer (2).

Notch signaling is a very important signaling pathway involved in many cellular regulations, such as cell proliferation, differentiation, development, and homeostasis (7, 10, 11). This pathway is activated when Notch receptors (Notch1-4) from neighboring cells get bound with their corresponding ligands (delta-like ligands 1, 3, and 4 and Jagged 1 and 2) (7). A two-step proteolytic cleavage occurs, governed by an ADAM family metalloprotease, tumor necrosis factor-a-converting enzyme (TACE). Then, the remaining portion, which consists of the intracellular and transmembrane domains, is cleaved by the γ-secretase complex (8). This enzymatic cleavage of the Notch protein creates what is known as the Notch intracellular domain (NICD). After that, NICD moves to the nucleus, where it binds to and converts a transcriptional repressor (the CSL/RBP-J complex) to an activator. Subsequently, it regulates gene expression (e.g., Hes1, p27cip1/waf1, cyclinD1, c-Myc, p21, Slug, and other genes), giving rise to imperative cellular processes (12).

Interestingly, it has been described that some malignancies, SCLC cells, and tumor tissues do not express Notch1 protein (13). For this reason, many studies have been going on to investigate the role of Notch1 signaling in different types of cancer. Some studies showed a tumor-promoting effect for Notch1 signaling and thus linked it to a poor prognosis in breast cancer (14), gastric cancer (15, 16), as well as NSCLC, and therefore proposed the possible use of Notch inhibitors in cancer treatment (17–19). In this article, we explored the role of Notch1 signaling in both in vitro and in vivo studies, focusing on various aspects of SCLC: proliferation, apoptosis, invasion, metastasis, epithelial-mesenchymal transition (EMT), and chemoresistance.

## Notch1 Expression in Lung Cancer

As different cancer types exhibit distinct characteristics, it’s expected that they do not share the same molecular pathways. Accordingly, to describe Notch1-related effects in SCLC, it is essential to build thorough knowledge about Notch1 and its related protein expression (Hes1, c-Myc, and Jagged1) in various lung cancer cell lines (Table 1).

To measure this, western blotting (WB) analysis was done on several cell lines, including SCLC (DMS53, H69, H69AR, H889, H1688, NCI-H209, and SBC3), ADC (A549, H358, and H1975), and SCC (H226, and H2170). All the SCLC cells (except H69AR and SBC-3) didn’t express the Notch1 transmembrane part and intracellular domain (NICD), while the expression was clearly detected in all the NSCLC (ADC and SCC) cell lines. Besides, both SCLC and NSCLC cells have shown positive Hes1 and Jagged1 expression (13, 20).

To further understand these findings, immunohistochemistry (IHC) was performed to assess the cellular localization of Hes1 and Jagged1 proteins in the lung cancer tissue samples obtained from 54 patients. These proteins were located in the nucleus in SCLC and SCC samples, yet cytosolic localization was found in ADC ones (13). It was also noted that Hes1 and c-Myc proteins positively related to Notch1 expression in SCLC cells. However, expression of these proteins in NSCLC wasn't affected by changing Notch1 expression (13). These results deduce the fundamental role of Notch1 signaling in lung carcinogenesis.

## Notch1 Signaling in Cell Proliferation

Although Isaac Newton was not a pathologist, his third law of motion holds true for normal cell growth, i.e., apoptosis is equal to and opposite of proliferation. Unfortunately, cancer cells do not obey this law! It possesses a collection of unique characteristics that give the cancer its “self-sufficiency” in growth and its ability to evade cell death (21). As has been mentioned, Notch signaling is one of the most important signaling pathways implicated in cell growth and carcinogenesis. To study effect of Notch1 on the cancerous cells, scientists used different technologies to alter its expression (22). Two of the most common techniques used are (1) small interfering RNA (siRNA), to down-regulate the expression of Notch1 (23), and (2) gene supplementation: cells transfected with a venus Notch1 intracellular domain (v.NICD) plasmid or with adenovirus as a vector (AdNotch), to increase Notch1 expression (24, 25). Knocking down (KD) Notch1 by siRNA in the H69AR cell line increases apoptotic signals, as indicated by the increase in expression of apoptotic markers caspase 3 and cleaved caspase 3 and a decrease in the expression of anti-apoptotic marker BCL-2. In addition, there was also a significant increase in the expression of p-H3 and pAkt proteins, which indicate high mitotic activity. Similarly, pAkt and p-H3 protein expression have also increased in the SBC-3 cell line treated with siRNA against Notch1, but the cells did not show a significant statistical difference in apoptotic signals (26). These observations state that inhibition of Notch1 signaling in these cell lines promotes a proliferative effect, which overwhelms the apoptotic activation.

On the other hand, inducing Notch1 expression in H69 and xenotransplanted H1688 cells by v.NICD showed an increase in apoptotic signals, indicated by the increase in the expression of cleaved caspase-3 protein and the decrease in the expression of BCL-2 protein. In addition, there was a decrease in the expression of p-H3 and pAkt proteins (26). This indicates that inducing Notch1 protein expression in these cell lines decreases cell proliferation and promotes apoptosis.

Furthermore, the use of recombinant adenovirus technology to induce Notch1 in DMS53 and NCI-H209 cells has demonstrated a significant decrease in the cell number compared to the control cells measured by the MTT assay (20). All the results stated above are summarized in Table 2.

**Table 1:** SCLC cell lines. Some important SCLC cell lines and their features including Notch1 expression.

**Cell line**	**Tissue of origin**	**Morphology**	**Notch1 expression**	**Other**	**References**
H69AR	Human lung	Epithelial	Yes	Multi-drug resistant, e.g., Adriamycin	(13)
SBC-3	Human lung	Epithelial	Yes	-	
H69	Human lung	Floating aggregates	No	Expresses insulin-like growth factor II receptor	
H1688	Human lung; derived from metastatic site: liver	Epithelial	No	Classic small cell lung cancer	
DMS53	Human lung	Epithelial	No	Expresses epidermal growth factor, transforming growth factor-β	(26)
NCI-H209	Human lung; derived from metastatic site: bone marrow	Tight floating cell clusters	No	-	

**Table 2:** Proliferation and apoptosis. The effect of Notch1 expression in proliferation and apoptosis in different SCLC cell lines. (↑) increase, (↓) decrease, (-) no change.

Cell line	Intervention	Proliferation	Apoptosis	Conclusion (Dominant result)	Reference
H69AR	siRNA (Knocking down)	↑↑↑	↑	Proliferation	(25)
SBC-3		↑↑↑	-		
H69	v.NICD (Induction)	↓↓↓	↑↑	Suppression	
H1668		↓	↑↑		
DMS53	AdNotch1 (induction)	↓↓↓	Not observed		(26)
NCI-H209		↓↓	Not observed		

**Table 3:** Invasion and metastasis; summarized from (52,53)

H69AR siNotch 1 & SBC-3 siNotch 1			
	WB^1^	RT-PCR^1^	IFA
E cadherin	Decreased	Decreased	Decreased
Snail	Increased	Increased	-
Slug	Increased	Increased	-
Vimentin	Increased	-	Increased
Twist	-	Increased	-
GL2	-	-	Increased
**H69 v.NICD**			
	WB^1^	RT-PCR^1^	IHC
E cadherin	Increased	Increased	Increased
Snail	Decreased	Decreased^2^	Decreased
Slug	Decreased	Decreased^2^	Decreased
Vimentin	No change	-	-
Twist	-	Decreased	-
GL2	-	-	Decreased

1 The experiment was performed in triplicate. 2 In *Hassan et al. (2014),* there was no change in these markers (52).

The results are compared to the control.

**Fig. 1:**
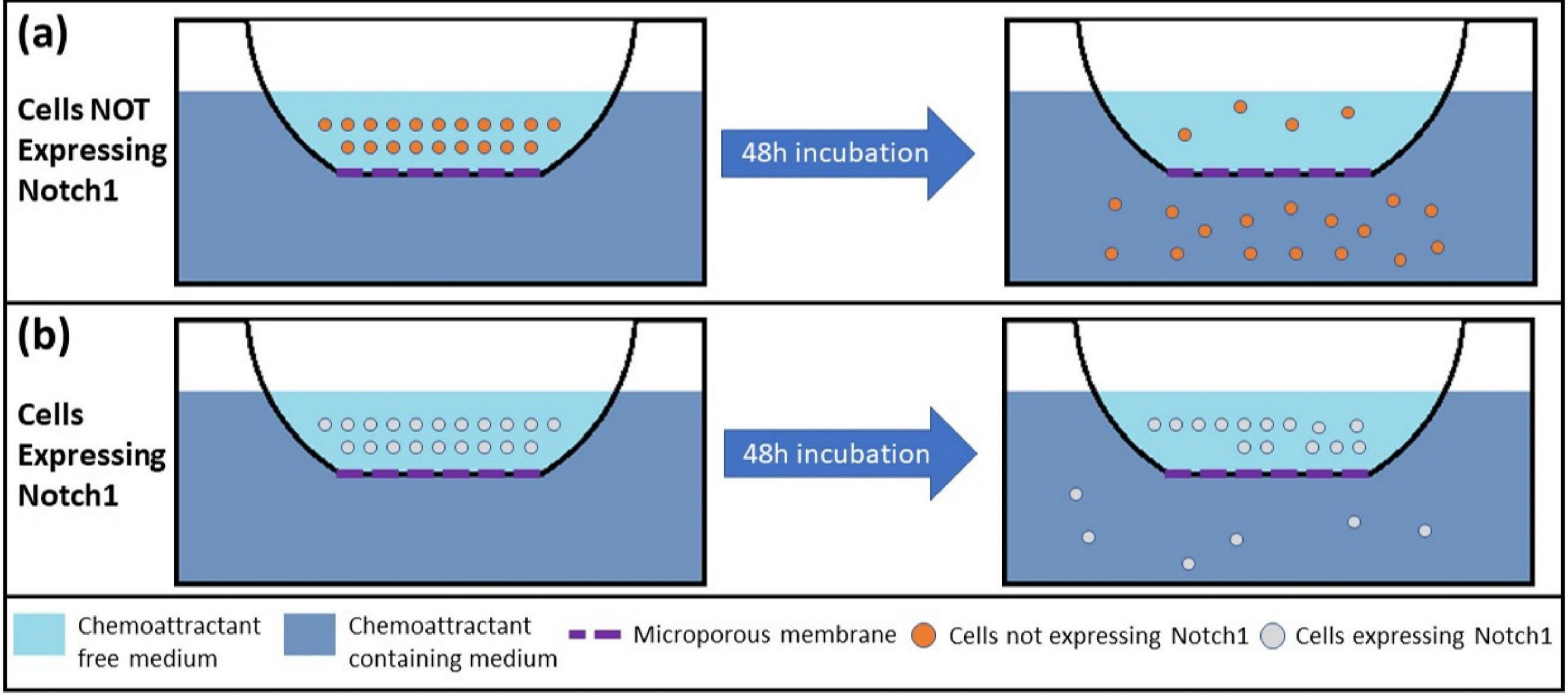
Matrigel Invasion Assay.

BioCoat Matrigel Invasion Chamber measures the invasion activity of different SCLC cell lines in relation to Notch1 expression. (a) Inoculation of cells not expressing Notch1; siRNA Notch1 transfected H69AR or SBC3 cells, or control H69 cells. (b) Inoculation of cells expressing Notch1; v.NICD transfected H69 cells, control H69AR, or control SBC3 cells. Notice that Notch1 expression in these SCLC cells demotes their invasion activity.

**Fig. 2:**
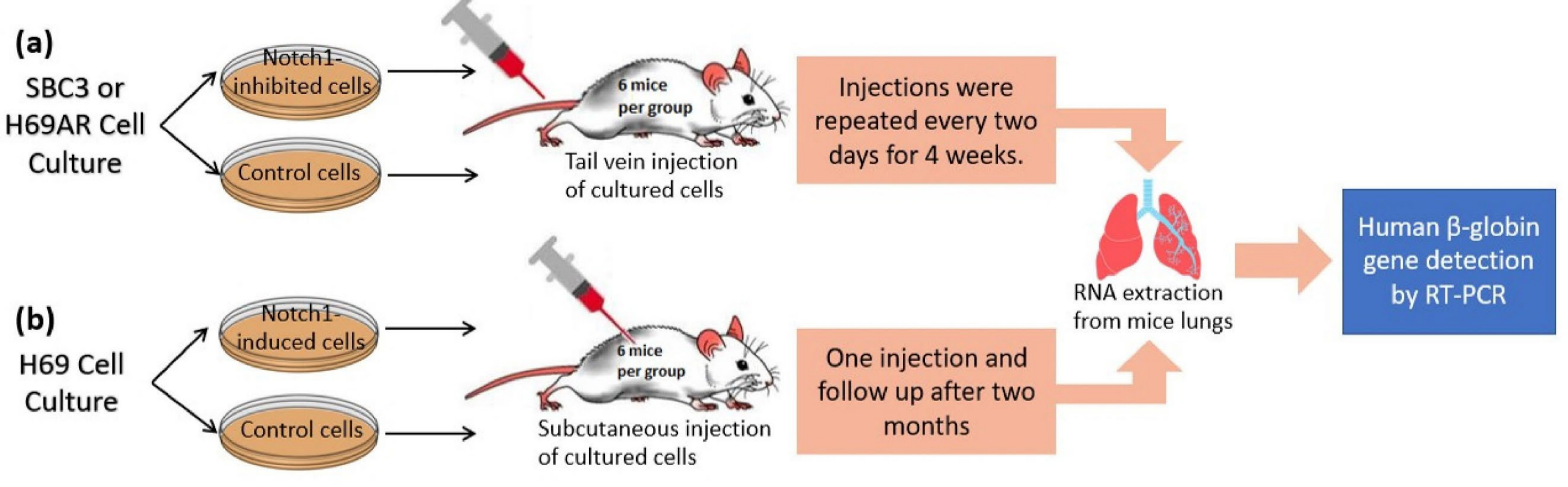
In vivo metastasis experiment.

Mice experiment assessing metastatic activity of different SCLC cell lines in relation to Notch1 expression. **(a) **Tail vein injection in a group of mice with siRNA Notch1 transfected SBC3 or H69 cells compared with its respective control group.** (b) **Subcutaneous injection in a group of mice with v.NICD Notch1 transfected H69 cells compared with its respective control group. The human β-globin gene was detected by RT-PCR after RNA extraction from mice lungs in the first and second experiments after 4 weeks and two months, respectively.

**Fig. 3:**
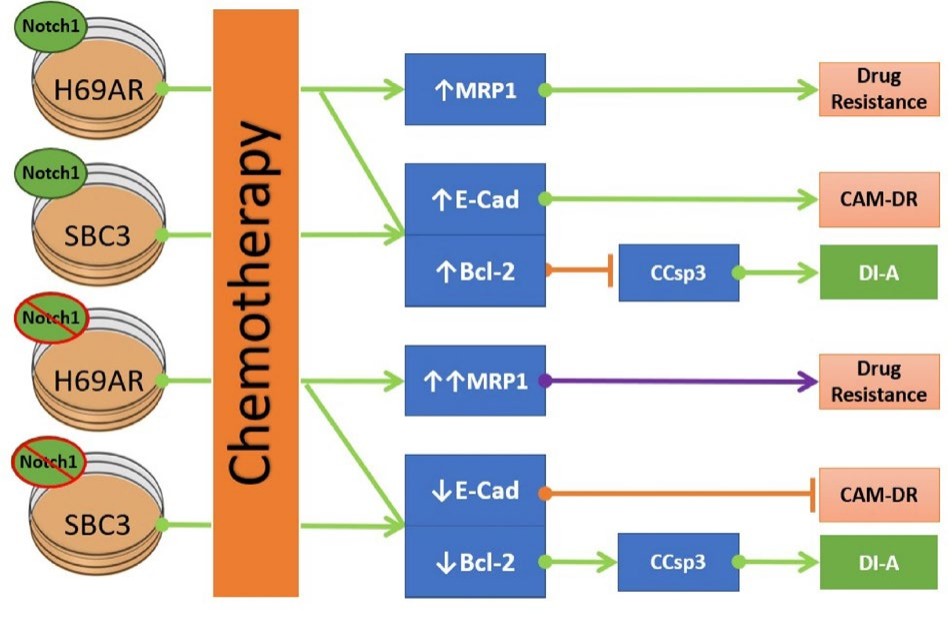
Chemoresistance in SCLC.

Role of Notch1 protein expression in chemoresistance of different SCLC cell lines. Multidrug resistance-associated protein-1 **(MRP-1)**, E-cadherin** (E-Cad)**, B-cell lymphoma2 **(Bcl2)**, cleaved caspase3 **(CCsp3)**, cell-adhesion-mediated drug resistance **(CAM-DR)**, drug-induced apoptosis **(DI-A)**. Note that MRP-1 is expressed strongly in H69AR SCLC cell lines. Notch1 signaling pathway is involved in the chemoresistance of SCLC.

**Fig. 4:**
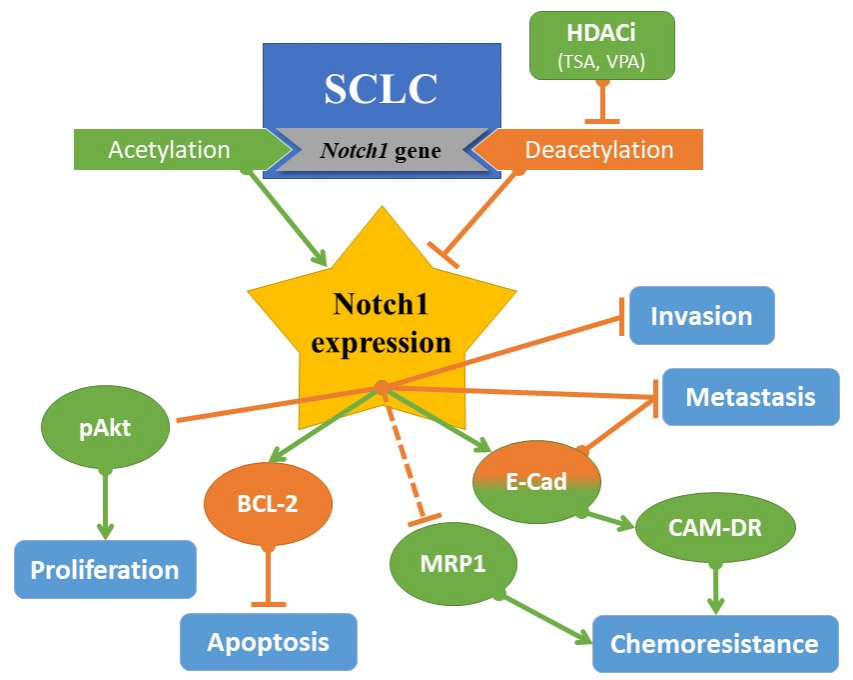
In vivo metastasis experiment.

Histone acetylation at the promoter region of *Notch1* increases Notch1 protein expression. This leads to subsequent alterations in cellular functions in favor of cancer suppression. Histone deacetylase inhibitor **(HDACi)**, trichostatin A **(TSA)**, valproic acid **(VPA)**, Multidrug resistance-associated protein-1 **(MRP-1)**, cell adhesion-mediated drug resistance **(CAM-DR)**.

Even though Notch1 protein expression showed promising results in inhibiting the growth of the SCLC, other studies on other cancer types demonstrated opposite results (27–32).

This indicates the diverse effects of the Notch signaling pathway and that its effect is dependent on the cell type (33).

A possible explanation for the mechanism of Notch1-induced growth inhibition in SCLC might be related to p21waf1/cip1 and p27kip1 induction. AdNotch1-infected NCI-H209 cell lines showed a significant increase in both genes, while in the DMS53 cell line, the increase was restricted to p21^waf1/cip1^. This suggests that Notch1-induced growth inhibition might be due to the induction of these tumor suppressor genes (20). In conclusion, Notch1 expression has a tumor suppressor role in the proliferation of SCLC cells.

## Histone Acetylation in Notch1 Expression

Histone acetylation is a covalent post-translational modification that, among others (methylation, phosphorylation, etc.), can impact gene expression by altering chromatin structure. DNA is wrapped around eight core histone proteins (two each of H2A, H2B, H3, and H4), forming a core structural subunit of chromatin known as the nucleosome. Primarily, histone H3 is acetylated at lysines 9, 14, 18, 23, and 56, while histone H4 is primarily acetylated at lysines 5, 8, 12, and 16 (34). Researchers have studied histone acetylation and its role in lung cancer progression (35–38). Several experiments have studied effect of treating lung cancer cells with histone deacetylase inhibitors (HDACi), like trichostatin A (TSA) (39–41), valproic acid (VPA) (42–44), and thailandepsin-A (45).

In Hassan *et al.* **(2017)**, correlation between histone acetylation and Notch1 protein expression in the lung carcinoma was investigated; the expression of acetylated histone H3 in the lung cancer cells was tested by WB and by chromatin immunoprecipitation quantitative PCR (ChIP-qPCR). In WB, the acetylated histone H3 was more often expressed in the SCLC compared to the NSCLC cells. In addition, the SCLC cells expressing Notch1 protein (SBC-3, H69AR) demonstrated higher fold enrichment levels of acetylated histone H3 around the Notch1 promoter compared to the non-expressing Notch1 protein SCLC cells (H69, H1688) in the ChIP-qPCR assay (46). Moreover, another experiment was done to further clarify the relation between histone acetylation and Notch1 protein expression in SCLC after treating the cells with trichostatin A (TSA) (47). First, the cells not expressing the Notch1 protein, H69 and H1688, were treated with TSA at 300 nM and 1000 nM, respectively. This resulted in an increase in expression of Notch1 by WB, immunofluorescence assay (IFA), and reverse transcriptase-polymerase chain reaction (RT-PCR). In addition, there was 30–50% cell growth inhibition in vitro. Likewise, TSA treatment increased acetylated histone H4, resulting in dose-dependent cell growth inhibition followed by apoptosis (47). Next, the level of histone H3 acetylation around the Notch1 promotor region was compared between the TSA-treated cell lines and untreated SCLC cells of the same cell lines using the ChIP assay. The results of this test showed an increase in histone H3 acetylation levels in TSA-treated H1688 cells but not for H69 cells, which may be due to the difference in TSA concentration levels used in the experiment. Lastly, the TSA-treated cells H1688 have regained Notch1 activities, which was denoted by the acquisition of epithelial-like tumor morphology after xenotransplantation as well as the “positive” change in biological properties: an increase in E-cadherin (E-Cad) epithelial markers for cell-cell adhesion, cleaved caspase 3 (CCsp3), and a decrease in both vimentin (Vim) and snail (mesenchymal transition markers), and phospho-histone H3 (P-H3), which are in favor of cancer suppression (46).

In addition, such restoration of Notch1 expression and the appearance of epithelial-like morphology in some areas highlight the possible role of epigenetic mechanisms, especially histone acetylation, in the histogenesis of combined SCLC (cSCLC), in which both SCLC and NSCLC components co-exist in same cancer (46).

Furthermore, valproic acid (VPA) is used as an antiepileptic drug, mood stabilizer, and HDAC inhibitor to alter histone acetylation. To justify its possible role in Notch1 full-length expression and activation, the SCLC (DMS53) cell line was treated by VPA. The results detected are as follows: First, there was a dose-dependent increase in the expression of full-length Notch1 and its related molecules, active NICD and Hes-1, as detected by WB with a concentration as minimal as 1 mM VPA. Second, histological studies indicated decreased cell density and increased differentiation of VPA-treated cells. Third, the MTT proliferation assay has shown significant dose-dependent inhibition of SCLC cell proliferation (48).

The results of the mentioned studies elucidate that use of HDAC inhibitors (TSA and VPA) could lead to histone acetylation of Notch1, leading to a higher Notch1 protein expression and subsequent suppression of SCLC growth.

## Notch1 Signaling in Invasion and Metastasis

One of the most important hallmarks of cancer cells is their ability to invade and metastasize (49). Thus, it’s important to investigate whether Notch1 signaling has a role in spreading SCLC. Cancer cells acquire these peculiar properties of invasion and metastasis through epithelial-mesenchymal transition (EMT). In this process, the epithelial cells lose their cell polarity, cell-cell adhesion, and attachment to their basement membrane, gaining the phenotype of mesenchymal cells (50). When this process is reversed, it’s called mesenchymal-epithelial transition, one of the cellular mechanisms of cancer treatment (51).

### In vitro Experiments

Experiments have been done to study the role of Notch1 signaling in invasion and metastasis using various techniques, i.e., WB, RT-PCR, IFA, and IHC. The data obtained from these experiments are summarized in Table 3.

After KD Notch1 in H69AR and SBC3 cell lines, the expression of E-cadherin (E-Cad), snail, slug, vimentin, twist, and gamma-laminin 2 chain alpha (GL2) markers were analyzed by WB, RT-PCR, and IFA. The results were as follows: there was a decrease in the expression of e-cadherin (detected by WB, RT-PCR, and IFA), an increase in the expression of snail, slug (detected by WB and RT-PCR), vimentin (detected by WB and IFA), twist (detected by RT-PCR), and GL2 (detected by IFA), stating that these Notch1-inhibited cells have undergone EMT and got an increase in their motility (52). Meanwhile, the expression of the same markers was analyzed by WB, RT-PCR, and IHC in the H69 cell line after the induction of Notch1 expression. The results were as follows: there was a remarkable increase in the expression of E-Cad (detected by WB, RT-PCR, and IHC) and a decrease in snail and slug expression (detected by WB, IHC, and RT-PCR). Also, there was a decrease in the expression of twist (detected by RT-PCR) and GL2 (detected by IHC). Vimentin expression did not show any significant change when detected by WB. These observations conclude that induction of Notch1 expression in these cells denoted EMT and enhanced cell-cell adhesion (52, 53).

### Epithelial-mesenchymal Transition (EMT)

Many studies have highlighted features of EMT in SCLC in many aspects, like decreased intracellular junctions, loss of apical-basal polarity (54, 55), and increased metalloprotease activity (56–59). Moreover, it was previously reported that SCLC cells can roll along the vessel wall, mimicking leukocytes (60). However, because SCLC cells lack prominent actin stress fiber formation and complete cell individualization, it cannot be considered a complete EMT-like cancer (50, 61). 

Achaete‐scute homolog 1 (ASCL1) is a proneural basic‐helix‐loop‐helix (b-HLH) transcription factor known for its pivotal role in NE differentiation and SCLC (62). The expression of ASCL1 is positively regulated by INSM1 (63). ASCL1 may also promote invasion and migration of cancer cells by altering Cdk5/p35 expression (64). Notch is a down-regulator of ASCL1 (65, 66). It's posted that the molecular pathway of small cell carcinogenesis may be due to reduced Notch1 activity, leading to an increase in the expression of ASCL1 (53), thus promoting EMT and NE differentiation in SCLC cells.

As shown in Table 3, Notch1 induction in SCLC cells induced features of epithelial differentiation by increasing the expression of the epithelial marker E-Cad and decreasing the expression of mesenchymal markers (snail, slug, and twist). On the other hand, inhibition of Notch1 signaling in these cells augments the EMT (52, 53).

In conclusion, induction of Notch1 expression in SCLC could minimize EMT and diminish the hallmarks of invasion and metastases, possibly through downregulation of ASCL1.

### Invasion Assay

BioCoat Matrigel Invasion Chamber is a technique used to assess ability of invasion of the cancer cells. In this technique, the tested cells are inoculated in one chamber, and a chemoattractant is put in the second chamber. A microporous membrane separates the two chambers. Number of the inoculated cells detected in the second chamber after a 48-hour incubation represents their invasion ability. This technique was used to assess the effect of Notch1 signaling on the invasion ability of the SCLC cells (Figure 1). Inhibition of Notch1 protein in H69AR and SBC3 cells increases the number of invading cells compared to the control cells (52, 53). In addition, induction of Notch1 expression in H69 cells decreases the invasive ability, compared to the control H69 cells (53).

### In vivo Experiments

Experiments using mice or other animals are always superior to lab tests when testing for a drug or a tumor character. This is because they better estimate what may happen inside the human body. Different SCLC cell lines were injected into different groups of mice to observe their ability to metastasize in relation to Notch1 protein expression.

In the first experiment, siRNA Notch1-transfected SBC3 cells were intravenously injected via the lateral tail veins of a six-mice group, while another group was injected by SBC3 cells as a control. The injections were repeated every two days and continued for four weeks. The lungs of the mice were removed, and the RNA was extracted to detect the human β-globin gene (a marker for metastatic human cancer cells in mouse tissue) by RT-PCR. The expression of this gene was significantly increased in Notch1-inhibited SBC3 cells compared to the control group (52). This difference in gene expression states that Notch1-inhibited cells have more metastatic potential.

In the second experiment, v.NICD-transfected H69 cells were subcutaneously injected into a group of six mice, and another control group of six mice was injected with H69 cells. Two months later, the human β-globin gene was detected after the extraction of RNA from mouse lung tissue. The expression of this gene was significantly decreased in Notch1-induced H69 cells compared to the control group (52). These different results in gene expression state that there is less metastatic potential in these Notch1-induced cells.

In the third experiment, Notch1-inhibited H69AR cells were tested in a similar manner to the first experiment of SBC-3 cells. However, the results of human β-globin gene expression have not shown any significant difference in this cell line (52). (Figure 2) shows a schematic representation of these experiments.

The results of the previously mentioned experiments significantly explicate that Notch1 expression in SCLC cell lines reduces the metastatic ability of these cancer cells.

It was noted in some studies that hypoxia induces Notch1 activity and subsequently increases EMT, along with suppressing the expression of E-cadherin in the cervical (C-33A), breast (MCF7), colon (HCT-116), ovarian (SKOV-3), and glioma (U-87MG) cell lines (67, 68). However, these studies have experimented with tumor cell lines other than SCLC. These differences in the effect of the Notch signaling pathway between cell lines illustrate the complexity of this pathway. Even with its diversity, all the mentioned studies have shown that the expression of Notch1 protein in SCLC switches off EMT, reduces cell motility, invasion, and metastatic ability, and enhances cell-cell adhesion.

## Notch1 Signaling in chemo-resistance

Cancer stem cells (CSC) are a special kind of cancer cell that possesses the ability to differentiate and self-renewal (69). This ability of CSC plays an important role in resistance to chemotherapeutic agents and, thus, the failure of conventional cancer therapy (70). It was previously reported that dysregulation of Notch1 signaling is one of the most important cellular pathways that give rise to CSC (71). Doxorubicin (Adriamycin) is a chemotherapeutic agent that can convert the drug-sensitive H69 SCLC cell line into the multi-drug-resistant (MDR) phenotype H69AR (72). Some of the factors involved in the acquisition of anti-cancer drug resistance, as well as the MDR conversion phenomena, include ATP-dependent transporters (e.g., MRP-1), which sense and expel anti-cancer drugs out of the cells (73), anti-apoptotic factors such as Bcl-2, which promotes cell survival and defends against drug-induced apoptosis (74, 75), the induction of drug detoxification mechanisms (75), and cell adhesion-mediated drug resistance (CAM-DR), in which chemotherapy-induced apoptosis is suppressed when cancer cells adhere to the extracellular matrix, increasing their resistance to chemotherapy (76).

Figure 3 summarizes the role of Notch1 protein expression in the chemo-resistance of different SCLC cell lines. First, the inhibition of Notch1 expression in the H69AR cell line increases the expression of MRP-1, increasing cancer chemo-resistance. On the other hand, the inhibition of Notch1 expression in both H69AR and SBC-3 cell lines (1) decreases E-Cad, thus reducing cell adhesion and switching off CAM-DR, and (2) decreases Bcl-2, leading to an increase in CCsp3 and thus drug-induced apoptosis (DI-A). These Notch1-inhibited cells were tested for their chemo-resistance and compared to control cells. In these tests, high concentrations of doxorubicin (10000 μM) were used in a 24-hour culture to measure cancer cells’ chemo-resistance abilities (77). It was found that SBC-3 cells with KD Notch1 show a significant reduction in cell number, indicating a loss of their chemo-resistance ability. However, Notch1-inhibited H69AR has not shown a statistically significant difference in the number of survival cells compared to their control. This might be explained by the increased expression of MRP-1, thus maintaining the chemo-resistance ability of H69AR cells (77). These findings point out a possible role of the Notch1 protein in the chemo-resistance of SCLC cells.

## Conclusion

The Notch1 signaling pathway is a crucial molecular system in regulating numerous cellular processes in various types of cancer. Despite all the rapidly accumulating knowledge, many questions remain without a clear answer. Why does the role of Notch1 vary in different cancer cells, even within the same cancer type? What other signaling components are associated with Notch1 signaling, and how do they affect its outcomes? What are the other Notch1 target genes, and how are they modulated?

This review presents a profound understanding of Notch1's role in the context of SCLC cells. It was proposed previously that Notch1 might be oncogenic for many cancer cells (13–18). However, this review illustrated that the expression of this signaling pathway suppresses SCLC cells by decreasing their proliferative capability and relatively increasing their apoptosis. Furthermore, it switches off metastasis and invasion and contributes to cell chemo-resistance. Also, HDACi (TSA and VPA) can be used as potent inducers of Notch1, which may be a potential therapeutic target for SCLC management (Figure 4). Notch1 expression in these cells induces an epithelial-like cell arrangement and could play a role in the origin of cSCLC. In a nutshell, the effect of such a sophisticated signaling pathway in tumor carcinogenesis should not be generalized to all human cancers, and further studies are needed to better tailor therapeutic plans based on the specific cellular context. 

## Figures Design

All the figures were designed by Microsoft 365; PowerPoint (version 2016) (Redmond, WA, US).

## Abbreviations

ASCL1: Achaete‐scute homolog 1, CSC: cancer stem cells, cSCLC: combined SCLC, EMT: epithelial-mesenchymal transition, HDAC: histone deacetylase, IFA: immunofluorescence assay, IHC: immunohistochemistry, KD: knockdown, MRP-1: Multidrug resistance-associated protein-1, NE: neuroendocrine, NICD: Notch Intracellular Domain, RT-PCR: reverse transcriptase polymerase chain reaction, siRNA: small interfering RNA, TSA: trichostatin A, VPA: valproic acid, WB: western blot.
